# Oligogenic basis of premature ovarian insufficiency: an observational study

**DOI:** 10.1186/s13048-024-01351-1

**Published:** 2024-02-03

**Authors:** Panpan Long, Le Wang, Hangjing Tan, Ruping Quan, Zihao Hu, Minghua Zeng, Ziheng Deng, Hualin Huang, Jonathan Greenbaum, Hongwen Deng, Hongmei Xiao

**Affiliations:** 1 Institute of Reproductive & Stem Cell Engineering, School of Basic Medical Science, Central South University, 88 Xiangya Road, Changsha, 410008 Hunan China; 2 Center of Reproductive Health, School of Basic Medical Science, Central South University, Changsha, China; 3 Biomedical Research Center, Hunan University of Medicine, Huaihua, China; 4 Reproductive Medicine Center, Department of Obstetrics and Gynecology, The Second Xiangya Hospital, Central South University, Changsha, China; 5 Center of Biomedical Informatics and Genomics, Deming Department of Medicine, Tulane University School of Medicine, New Orleans, LA USA

**Keywords:** Whole-exome sequencing, Gene-burden analysis, Oligogenic inheritance, Premature ovarian insufficiency, ORVAL platform

## Abstract

**Background:**

The etiology of premature ovarian insufficiency, that is, the loss of ovarian activity before 40 years of age, is complex. Studies suggest that genetic factors are involved in 20–25% of cases. The aim of this study was to explore the oligogenic basis of premature ovarian insufficiency.

**Results:**

Whole-exome sequencing of 93 patients with POI and whole-genome sequencing of 465 controls were performed. In the gene-burden analysis, multiple genetic variants, including those associated with DNA damage repair and meiosis, were more common in participants with premature ovarian insufficiency than in controls. The ORVAL-platform analysis confirmed the pathogenicity of the *RAD52* and *MSH6* combination.

**Conclusions:**

The results of this study indicate that oligogenic inheritance is an important cause of premature ovarian insufficiency and provide insights into the biological mechanisms underlying premature ovarian insufficiency.

**Supplementary Information:**

The online version contains supplementary material available at 10.1186/s13048-024-01351-1.

## Background

Premature ovarian insufficiency (POI), characterized by the decline of ovarian function before 40 years of age, affects 3.7% of women globally [1]. It is highly heterogeneous, ranging from ovarian dysgenesis and primary amenorrhea to post-pubertal secondary amenorrhea, with elevated serum gonadotropin levels and hypoestrogenism [2]. Long-term complications include osteoporosis, cardiovascular/neurological disease, and cancer. The development of POI in patients with secondary amenorrhea can be a gradual process that encompasses occult, biochemical and overt stages [3]. Because of the irreversible nature of the decline in ovarian function, there is no effective method of restoring and improving ovarian function. Therefore, researchers should focus on early recognition, early diagnosis, and early intervention in disease development in patients with POI, which will subsequently be of great significance in improving the quality of life of patients in the near future and preventing complications in the long term. Its etiology is complex, involving genetic, immune, infectious, and iatrogenic factors [4]. Genetic factors have been identified in 20–25% of cases [5].

Whole-exome sequencing has been used to identify novel candidate genes for POI associated with various biological functions [6]. However, no genes have been implicated in more than 5% of cases, except for *BMP15*, *FMR1*, and *NOBOX* [7]. Genome-wide association studies (GWASs) have identified several single-nucleotide polymorphisms [8–15]. However, the cohorts were small, the results varied among populations, and the suggested candidate genes tend to lack biological evidence of a direct association with the ovary. Although whole-exome sequencing and GWASs have revealed a part of the genetic basis of POI, > 50% of cases are idiopathic [5]. The delayed intervention is a consequence of the failure to diagnose at an earlier stage [3]. Genetic testing can provide families with important information about the risks and etiology of POI [16]. None of the mutations in one or a few genes or a particular genetic mechanism explains most of the pathophysiological mechanisms of POI, and some of the challenges that have arisen in genetic studies have not yet been plausibly explained (e.g., despite familial onset of the disease, majority of patients present with sporadic cases, and some candidate genes are incomplete in families with autosomal dominant inheritance), suggesting the need to look at the genetic background of POI development from a new genetic perspective, which suggests that new causative mechanisms remain to be explored. The exploration of therapeutic targets, such as in vitro activation, holds significant importance in the context of research and providing guidance for genetic counseling and pregnancy planning [16].

**Fig. 1:**
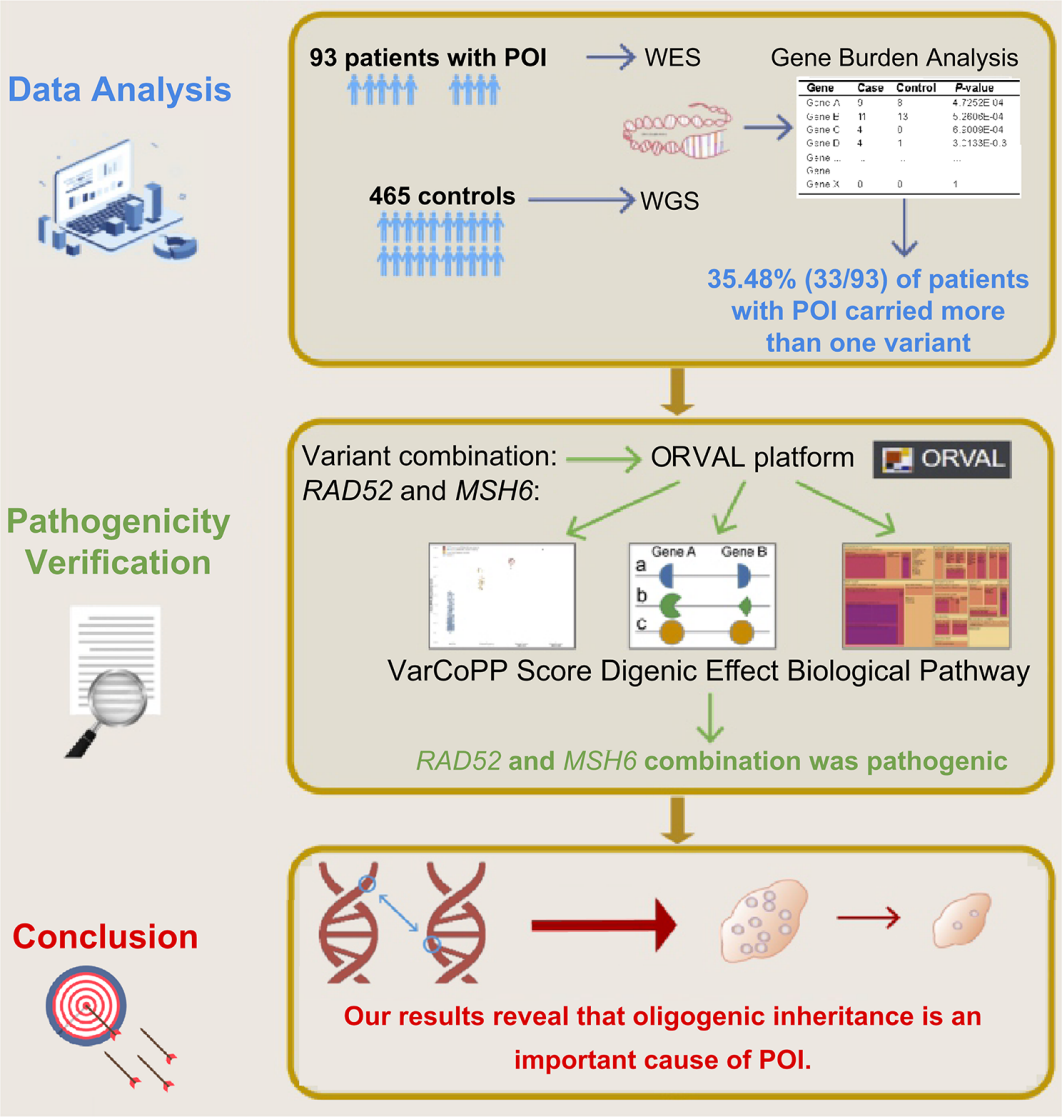
Overall study design. WES, whole-exome sequencing; WGS, whole-genome sequencing; POI, premature ovarian insufficiency

The inheritance of a trait (or disease) by a few genes is defined as oligogenic inheritance. It is an intermediate state between monogenic and polygenic inheritance [17]. Since the first report of retinitis pigmentosa as a digenic disease in 1994 [18], 207 digenic or oligogenic diseases have been reported [19]. Several studies [20–22] have reported the possibility of oligogenic inheritance in POI. The oligogenic inheritance pattern may be a more plausible explanation for the differences in clinical symptoms, time of onset, and severity of clinical manifestations among patients with POI. Variants at different loci in the same gene, or multiple genes with multiple variants, may contribute to the different clinical phenotypes of patients with POI. However, these studies mainly included patients with sporadic disease in European countries and did not include healthy control groups. Moreover, the authors did not validate their findings or investigate the potential mechanisms. Combinations of variants may cause POI by similar or different mechanisms and pathways. Therefore, we recruited Chinese individuals with POI and normal women (as controls) and aimed to investigate the oligogenic basis of POI, which may aid early diagnosis and treatment. The overall study design is shown in Fig. 1.

## Results

### POI cohort gene-burden analysis

Sequencing analysis of 93 patients with POI and 465 controls was performed. Gene-burden analysis was performed after whole-exome sequencing, quality control, and variant annotation. Genes were the basic study unit, and different groups were analyzed separately from controls. There were 7,549 variants, including 4,631 loss-of-function (included frame-shift, splice-site or stop variants) and 4,471 missense variants. In total, 2,924 variants, including 1,792 loss-of-function and 1,704 missense variants were significant in the comparison between patients and controls (*P* < 0.05; Additional File 1). The *P-*values and quantiles (calculated by ranking the genes according to *P-*values) are summarized in Additional File 2.

### Participants heterozygous for multiple variants

Regarding the 191 POI-related genes, 35.5% (33/93) of patients with POI and 8.2% (38/465) of controls were heterozygous for more than one variant (odds ratio, 6.20 [95% confidence interval: 3.60–10.60]; *P* = 1.50 × 10^− 10^; Table 1). Overall, multiple variants were more common in patients with POI than in controls.

**Table 1:** Number of POI-related variants in patients with POI and controls

	Patients with > 1 variant (*n*)	Odds ratio (95% confidence interval)	*P*-value
POI (*n* = 93)	33	1	/
Controls (*n* = 465)	38	6.20 (3.60–10.56)	1.50 × 10^–10^

POI, premature ovarian insufficiency

Additional File 3 provides an overview of the 33 patients with POI who were heterozygous for more than one variant. The proportions of patients who were heterozygous for two, three, four, and five variants were 16.1% (15/93), 10.8% (10/93), 7.5% (7/93), and 1.1% (1/93), respectively. The highest proportion of patients with POI was heterozygous for two variants.

### Analysis and validation of variant combinations

#### Gene-burden analysis

Among 191 POI-related genes, the top 15 genes (*P* < 0.05) ranked using gene-burden analysis are listed in Table 2. *RAD52* (*P* = 5.28 × 10^− 4^) and *MSH6* (*P* = 5.98 × 10^− 4^) were enriched in patients with POI, ranking first and second, respectively. We identified *RAD52* variants in 9/93 (9.7%) patients with POI; seven of these patients (77.8%) were heterozygous for an additional variant in a POI-related gene (*MSH6*, *TEP1*, *POLG*, *MLH1*, or *NUP107*; Additional File 4).

**Table 2:** Gene-burden analysis of POI-related genes

Gene	Patient	Control	*P*-value	Odds ratio (95% confidence interval)
***RAD52***	9 (9.7%)	8 (1.7%)	5.28 × 10^–4^	6.12 (2.30–16.31)
***MSH6***	11 (11.8%)	13 (2.8%)	5.98 × 10^–4^	4.66 (2.02–10.77)
***AR***	4 (4.3%)	0 (0%)	7.31 × 10^–4^	Inf (Nan–Inf)
***TP63***	4 (4.3%)	1 (0.2%)	3.18 × 10^–3^	20.85 (2.30–188.78)
***IGSF10***	6 (6.5%)	6 (1.3%)	7.26 × 10^–3^	5.28 (1.66–16.74)
***POLG***	4 (4.3%)	2 (0.4%)	8.33 × 10^–3^	10.40 (1.88–57.67)
***TEP1***	5 (5.4%)	4 (0.9%)	8.39 × 10^–3^	6.55 (1.72–34.87)
***MLH1***	6 (6.5%)	7 (1.5%)	1.17 × 10^–2^	4.51 (1.48–13.75)
***ERCC6***	4 (4.3%)	3 (0.6%)	1.70 × 10^–2^	6.92 (1.52–31.46)
***FANCG***	2 (2.2%)	0 (0%)	2.75 × 10^–2^	Inf (Nan–Inf)
***MSH5***	2 (2.2%)	0 (0%)	2.75 × 10^–2^	Inf (Nan–Inf)
***GALT***	2 (2.2%)	0 (0%)	2.75 × 10^–2^	Inf (Nan–Inf)
***GDF9***	4 (4.3%)	4 (0.9%)	2.96 × 10^–2^	5.18 (1.27–21.10)
***FANCM***	3 (3.2%)	2 (0.4%)	3.48 × 10^–2^	7.75 (1.27–46.84)
***NUP107***	3 (3.2%)	2 (0.4%)	3.48 × 10^–2^	7.75 (1.27–46.84)

POI, premature ovarian insufficiency

#### Validation of the RAD52 and MSH6 combination

Two patients were heterozygous for both variants; the *RAD52* and *MSH6* combination was not detected in the control group (*P* = 0.027; Additional file 5). Oligomeric network analysis using the ORVAL platform showed that *RAD52* and *MSH6* existed in combination with candidate disease-causing variants (Additional File 6). In CADD raw score generation, gene haploinsufficiency prediction, and biological process similarity, VarCoPP predicted that the *RAD52* and *MSH6* combination was pathogenic (scores of 1.0; Table 3). Using the Digenic Effect predictor, loci were classified as “true digenic” or “monogenic + modifier” (Table 3 and Additional File 6).

**Table 3:** Pathogenicity and Digenic Effect prediction

Sample ID	Gene	Alleles	VarCoPP			Digenic Effect			
			Prediction score	Predicted class	Confidence zone	True digenic	Monogenic + modifier	Dual molecular diagnosis	Predicted class
64	*RAD52*	NC_000012.11 (NM_134424.4) c.1037 C > A	1.0	Disease-causing	99.9%	0.42	0.35	0.23	True digenic
	*MSH6*	NC_000002.11 (NM_000179.3) c.4068_4071dupTTGA							
66	*RAD52*	NC_000012.11 (NM_134424.4) c.1037 C > A	1.0	Disease-causing	99.9%	0.34	0.44	0.22	Monogenic + modifier
	*MSH6*	NC_000002.11 (NM_000179.3) c.3488 A > T							

#### Protein–protein interaction (PPI) networks

**Fig. 2:**
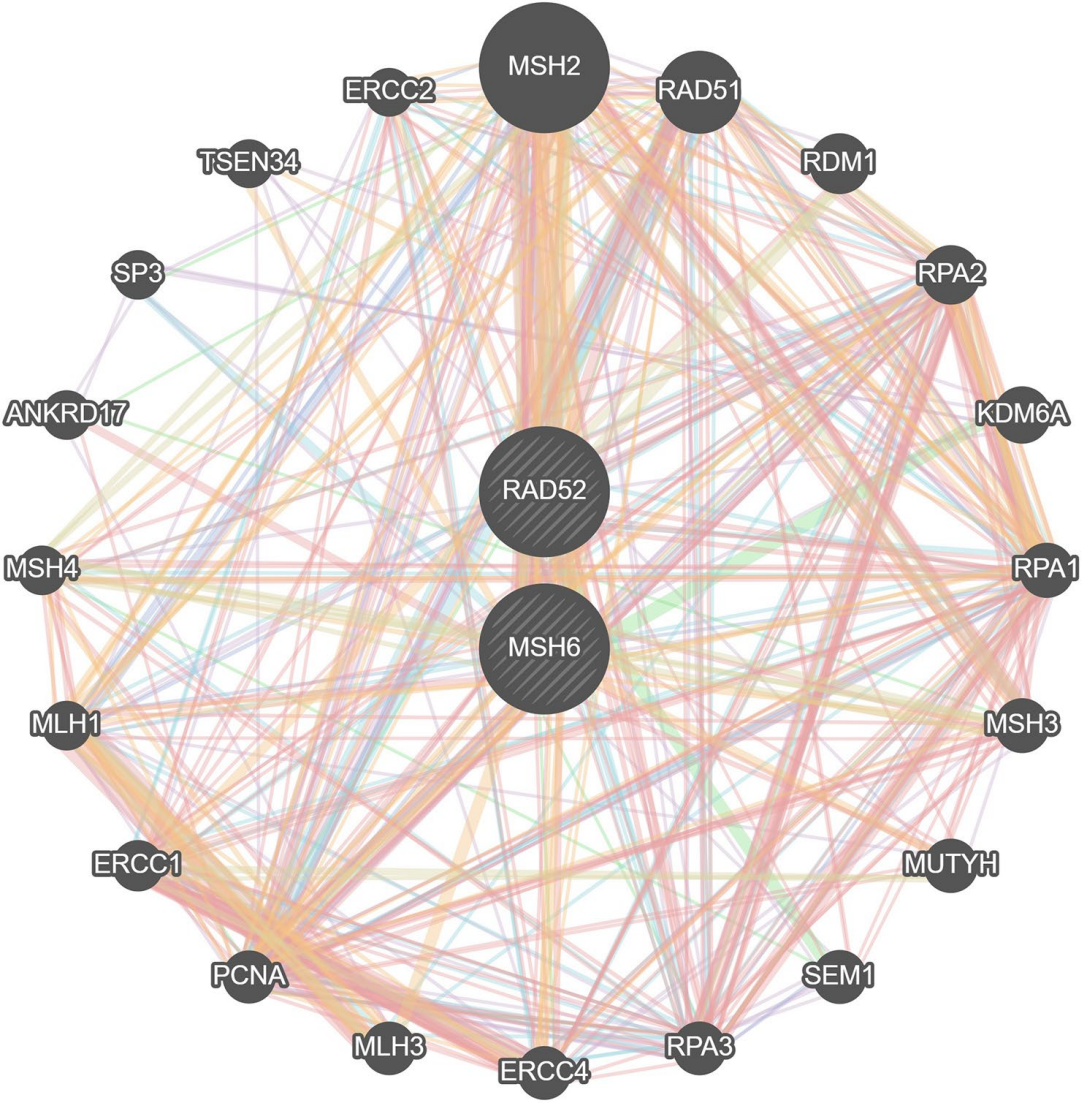
Protein–protein interaction (PPI) networks of RAD52 and MSH6.

PPI network analysis revealed associations of RAD52 and MSH6 with DNA damage-repair processes (such as DNA recombination, DNA repair complex, nucleotide-excision repair, double-strand break repair, and the homologous recombination pathway), suggesting their significant roles in DNA damage-repair processes (Fig. 2).

### Biological functions of POI-related genes

POI-related genes are involved in various biological functions, such as meiosis and DNA damage repair, gonadal formation, and ovarian development, and the encoded proteins serve as signaling molecules and transcription factors. Gene-burden analyses were performed on four gene sets with different biological functions. A significant difference was observed in the number of genes associated with meiotic and DNA repair pathways between patients with POI and controls (*P* = 4.04 × 10^–9^; Table 4).

**Table 4:** Patients heterozygous for variants in genes of different biological pathways

	Patients with > 1 variant (n)		Odds ratio (95% confidence interval)	*P*-value
	POI	Controls		
Meiosis and DNA repair	21	15	8.75 (4.31–17.76)	4.04 × 10^–9^
Gonadal formation	0	0	/	1
Ovarian development	5	3	8.75 (2.05–37.28)	0.004
Signaling molecules and transcription factors	5	2	13.15 (2.51–68.87)	0.001

POI, premature ovarian insufficiency

## Discussion

Whole-exome and -genome sequencing are widely used for the diagnosis and molecular analysis of POI, thereby gradually improving our understanding of its molecular basis. In the gene-burden analysis, multiple genetic variants and genetic variants associated with DNA damage repair and meiosis were more commonly found in patients with POI than in controls. The ORVAL-platform analysis supported the pathogenicity of the *RAD52* and *MSH6* combination. Our results indicate that oligogenic inheritance is an important cause of POI.

Regarding POI-related genes, the incidence of POI in patients with multiple variants was significantly higher than that in controls. However, some individuals in the control group also had multiple POI-related genetic variants. It is possible that variants on different alleles have different degrees of pathogenicity as some alleles may not be sufficient to cause a phenotypic trait or disease. Alternatively, one or more variants can cause POI, whereas simultaneous variants can mitigate or counteract the pathogenicity of the variant via interaction effects [23]. In this study, the *RAD52* and *MSH6* combination was classified as “true digenic” in one patient and as “monogenic + modifier” in another. *RAD52* variants were at the same locus in both patients, whereas *MSH6* variants were at different loci. This illustrates that the same gene combination with different alleles can lead to a different classification, indicating the complexity of predicting and classifying oligogenic pathogenic combinations [24]. In both patients, another variant was detected in addition to the *RAD52* and *MSH6* combination. However, it was difficult to predict the pathogenicity of combinations involving three or more variants. Additional data are needed to support the classification criterion of the *RAD52* and *MSH6* combination.

**Fig. 3:**
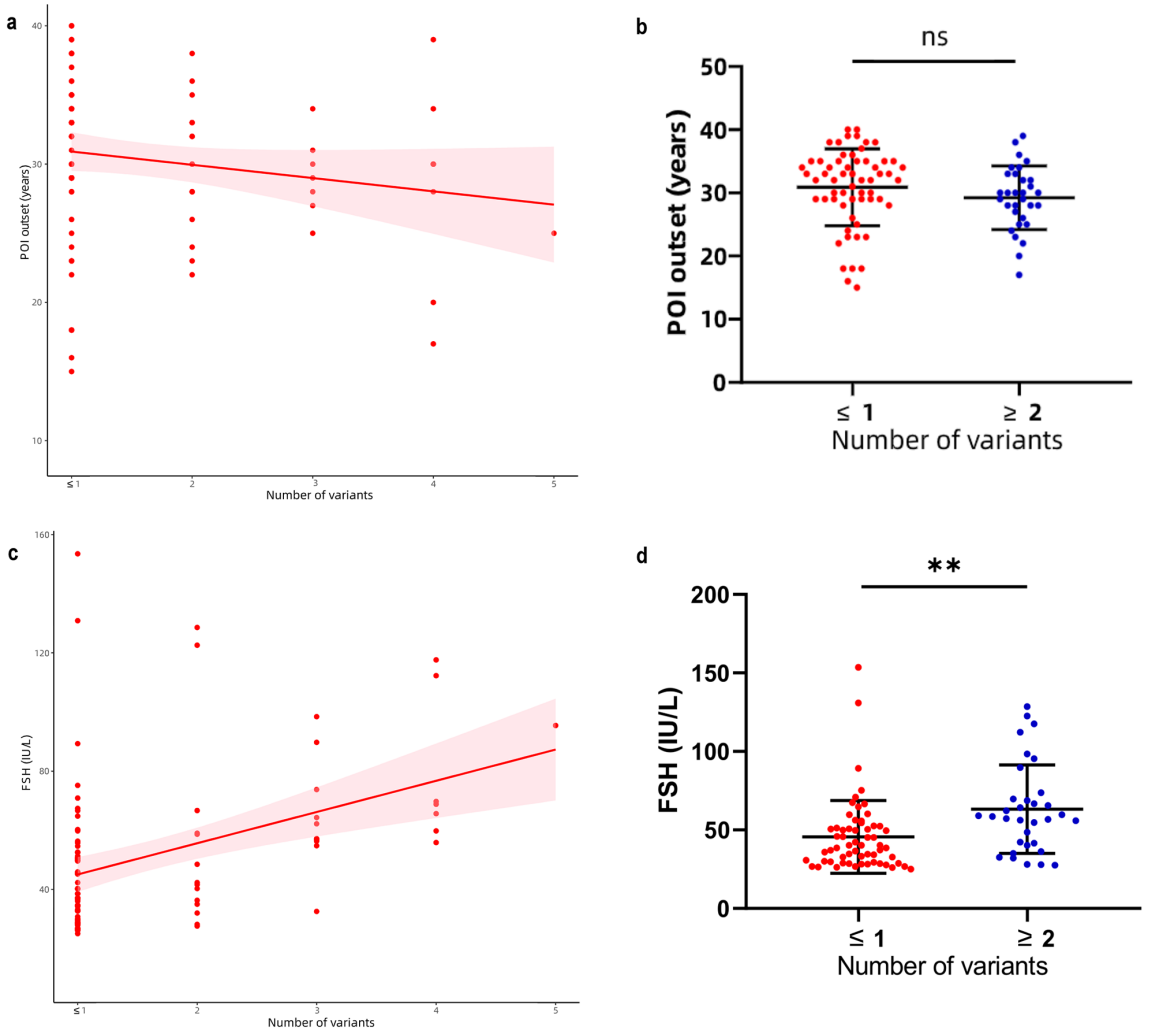
Relationship of age of onset, FSH and number of variants carried in patients with POI. (**a**) Relation between age of POI onset and the number of variants carried, (**b**) Comparison of age of POI onset in patients with one or no variants compared with those with two or more variants, (**c**) Relation between FSH and the number of variants carried in patients with POI, (**d**) Comparison of FSH in patients with one or no variants compared with those with two or more variants. FSH: follicle-stimulating hormone; ns: no significance; **P < 0.01

Using age of onset and FSH values as indicators of the severity of the POI phenotype, we analyzed the relationship between the age of onset (age at which oligomenorrhea or amenorrhea occurs) and FSH values of patients respectively, and the number of variants carried by patients with POI. Our results showed that a higher number of variants in patients with POI was associated with a lower age of onset (Fig. 3a), however, there were no statistical differences between groups (Fig. 3b); In addition, our results indicated a positive correlation between the value of FSH and the number of variants carried by patients with POI (Fig. 3c), and differences between groups were statistically significant (*P* < 0.01) (Fig. 3d). However, this analysis did not include factors such as the pathogenicity of variants and the contribution of different genes. More cases and data analyses are required to construct disease prediction models for POI, including age of onset, age at menopause, phenotype severity, master genes, and the specific relative contribution of each locus.

POI-related genes are involved in various biological functions, such as meiosis and DNA damage repair, gonadal formation, ovarian development, and the encoded proteins serve as signaling molecules and transcription factors [4]. *RAD52* is involved in DNA double-strand break repair and mitotic/meiotic recombination [7]. *RAD51*, which is the known causative gene for POI [25], is in the same gene family as *RAD52*. *MSH6* variants have been reported in patients with POI [26]. Although *RAD52* variants were more commonly found in patients with POI than in controls, *RAD52* has not yet been identified as a pathogenic gene for POI, and a clear pathogenic mechanism by which *RAD52* causes POI has not been determined. We found *TEP1* and *FANCG* as novel potential candidate disease-causing genes for POI. TEP1 is a part of the telomerase ribonucleoprotein complex that catalyzes the addition of new telomeres to chromosome ends [27]. A *FANCG* mutation has been identified in patients with Fanconi anemia of complementation group G [28]. The N-terminal nuclear localization signal of FANCA is necessary for FANCG binding, FANCC binding, sensitivity of complementary FAA lymphocytes to mitomycin C, and nuclear localization [28]. More evidence on whether *TEP1* and *FANCG* are causative genes for POI is required. Our results confirmed that the frequencies of genetic variants associated with the meiotic and DNA repair pathways differed between patients with POI and controls. Several variants are involved in the meiotic and DNA repair pathways. Therefore, these pathways represent promising therapeutic targets.

Whole-exome sequencing is a molecular diagnostic approach that can be used to explore multiple genetic variants. In an whole-exome sequencing analysis of 2,076 participants, 101 (4.9%) were found to be heterozygous for multiple genetic variants [29]. Oligogenic inheritance models have been proposed for 207 diseases in the OLIDA database. Tools for oligogenic analysis include DiGePred, OligoPVP, and VarCoPP/ORVAL. DiGePred is a random forest classifier developed to specifically identify pathogenic variant combinations based on biological networks, along with genomic, evolutionary, and functional data. DiGePred facilitates identification of genetic factors for rare non-monogenic diseases by scoring the potential of variant combinations to cause a biallelic disease [30]. OligoPVP combines a random forest classifier and a deep neural network to predict the pathogenicity of combinations of genetic variants, using feature sets from different tools (e.g., CADD and DANN) to classify the variant combinations as pathogenic or non-pathogenic [31]. In addition, VarCoPP is a machine-learning tool that identifies pathogenic variant combinations in gene pairs (digenic/bilocus variant combinations), generating classification scores for combinations of genetic variants using 11 different biological traits [32]. ORVAL is another tool that extends VarCoPP predictions to include more features (e.g., network-based exploration) [33] and integrates different resources to predict combinations of oligogenic variants that cause disease. Several studies have used VarCoPP/ORVAL to predict variant combinations [34–38], as in this study. The advantages of ORVAL over other tools include the number of predicted combinations of pathogenic variants, ability to classify combinations of variants, availability of detailed information on genes at the level of functional pathways, and visual presentation of the results. In this study, we investigated the oligogenic inheritance of POI using gene-burden analysis, quantified the contributions of variants to the disease by *P*-value ranking, and demonstrated that the frequency of multiple POI-related genetic variants differed significantly between patients with POI and controls. However, pathogenicity prediction was not possible using gene-burden analysis. Accordingly, this approach was combined with VarCoPP/ORVAL to demonstrate the roles of multiple POI-related genetic variants in the disease and to score and classify combinations of variants in patients for pathogenicity prediction. These findings support an oligogenic inheritance model of POI and will inform oligogenic studies in more disease cohorts.

In this study, 93 participants with POI and 465 controls were recruited. The sample sizes are being expanded and data from parents and other family members are being collected to verify whether the variants are consistent with familial co-segregation.

## Conclusions

Overall, we demonstrated that POI is consistent with an oligogenic inheritance model by going beyond the traditional methods of screening and validation of pathogenic genes for POI. Our findings will inform cohort-based oligogenic studies and provide insights into the biological mechanisms underlying POI. Our findings provide support for further research on the etiology of and potential therapeutic targets for POI.

## Methods

### POI group

In total, 93 patients with POI (Two and 91 displayed primary or secondary amenorrhea, respectively) were recruited by stage from Xiangya Second Hospital of Central South University, Hunan, China; Changsha Reproductive Medicine Hospital, Changsha, China; and Changsha Jiangwan Maternity Hospital, Changsha, China. All patients with POI were women aged < 40 years with oligomenorrhea or amenorrhea for ≥ 4 months and elevated follicle-stimulating hormone levels > 25 IU/L on two occasions > 4 weeks apart. Patients with chromosomal abnormalities, *FMR1* variants, pelvic surgery, endometriosis, ovarian infection, radiotherapy or chemotherapy, and endocrine autoimmune diseases were excluded. Blood samples (2–3 mL) were collected in EDTA anticoagulation tubes. DNA was extracted and stored at − 80 °C.

### Control group

In total, 465 women aged 45–65 years in the menopause stage (no amenorrhea and regular menstrual cycles before age 40) were recruited from The Third Affiliated Hospital of Southern Medical University in 2017. Whole-genome sequencing data were collected previously [39].

### Whole-exome sequencing and variant calling

In total, 191 POI-related genes were obtained from the literature [6, 7, 40] and IDDB database (Additional File 7). To clarify the role of genes with different biological functions in POI etiology, the biological functions are mainly categorized into meiosis and DNA damage repair, gonadal formation, ovarian development, and signaling molecules and transcription factors according to previous literature [6, 41]. We have organized the different gene sets based on four different biological functions (Additional File 8).

Whole-exome sequencing was performed in 93 patients with POI. Genomic DNA was extracted from peripheral blood. Whole-exome sequences were captured using the SureSelect Target Enrichment System for Illumina Paired-End Sequencing Library (Agilent Technologies, Santa Clara, CA, USA). DNA sequencing was performed on the Illumina HiSeq Platform (Illumina, San Diego, CA, USA). Reads were mapped to the GRCh37 genome. Variants were annotated using GATK, ANNOVAR, and custom pipelines.

### Gene-burden analysis

Gene-burden analysis of 191 POI-related genes was performed. The following filtering thresholds were applied: read depth, > 20; minor allele frequency, < 5% in GnomAD/ExAC/1000 Genomes Phase 3; and *in silico* prediction tools (REVEL, > 0.5; Splice Site, > 0.6; and scSNV, > 0.6). Genes were weighted using SKAT-O, and associations between genetic variants and POI were evaluated.

### Prediction of oligogenic pathogenic variant combinations

ORVAL is a web-based bioinformatics platform for predicting pathogenic variant combinations. VarCoPP, the variant combination pathogenicity predictor, was used to obtain the probability of whether a particular combination of pathogenic loci was a true positive result. The Digenic Effect predictor was used to classify pathogenic variant combinations as “true digenic,” “monogenic + modifier,” or “dual molecular diagnosis [32].”

### PPI networks

The GeneMANIA database (http://genemania.org/), a powerful tool for analyzing gene lists, predicting gene function, and prioritizing genes for functional analysis, was used to construct PPI networks for the genes. The database identifies genes with similar functions and assigns a value to each functional genome dataset based on the predicted value of the query by integrating multiple genomic and proteomic data. This integrated approach enhances the accuracy of gene function prediction and facilitates a deeper understanding of the complex interactions between genes within biological pathways.

### Statistical analysis

Gene-burden analysis was performed using “SKAT” (R version 2.2.4; R Foundation for Statistical Computing, Vienna, Austria). Fisher’s exact test was performed using “SciPy” (Python version 1.7.0) [42]. GraphPad Prism 8 (GraphPad Software, San Diego, CA, USA) was used for statistical analysis. *P* < 0.05 was considered statistically significant.

### Electronic supplementary material

Below is the link to the electronic supplementary material.

Additional file 1: **TableS1**. Gene-burden analysis of the POI cohort.

Additional File 2: **Figure S1**. Quantile-quantile plot. LOF, loss-of-function.

Additional File 3: **TableS2**. Cases involving more than one POI-related variant.

Additional File 4: **Table S3**. *RAD52* variants detected in patients with POI.

Additional File 5: **Table S4**. Patients heterozygous for the *RAD52* and *MSH6* combination.

Additional File 6: **Figure S2**. Oligogenic combination networks and radar plots. **(a, b)** Oligogenic combination networks for patients 64 and 66. Gene pairs are connected if they contain at least one pathogenic variant combination. Edge color: highest pathogenicity score (highest VarCoPP score) for a variant in the pair, shown from low (yellow) to high (dark red) pathogenicity scores. **(c, d)** Radar plots of the prediction results of the Digenic Effect for patients 64 and 66.

Additional File 7: **Table S5**. Gene sets included in the analysis

Additional File 8: **Table S6** Four gene sets with biological functions related to POI.

## Data Availability

The datasets used and/or analysed during the current study are available from the corresponding author on reasonable request.

## Abbreviations

ESHRE: European Society for Human Reproduction and Embryology
GWAS: Genome-wide association study
POI: Premature ovarian insufficiency
