# Supplementary material for: Oligogenic basis of premature ovarian insufficiency: an observational study
Source: J Ovarian Res. 2024 Feb 3;17:32. doi: 10.1186/s13048-024-01351-1 (PMC10837925; doi:10.1186/s13048-024-01351-1)
Supplement: Supplementary file 1 — Additional file 1: TableS1. Gene-burden analysis of the POI cohort. [file 13048_2024_1351_MOESM1_ESM.docx]

**Additional File 1**

**Table S1**. Gene-burden analysis of the POI cohort

| Patients (*n*) | Controls (*n*) | Variant group | *n* | *P-*value (SKAT-O) | |
| --- | --- | --- | --- | --- | --- |
|  |  |  |  | *P* < 0.05 | *P* < 0.01 |
| 93 | 465 | All | 7,549 | 2,924 (0.387) | 933 (0.124) |
|  |  | Loss-of-function | 4,631 | 1,792 (0.387) | 434 (0.094) |
|  |  | Missense | 4,471 | 1,704 (0.381) | 477 (0.107) |

POI, premature ovarian insufficiency.
