# Supplementary figures and images for: Oligogenic basis of premature ovarian insufficiency: an observational study
Source: J Ovarian Res. 2024 Feb 3;17:32. doi: 10.1186/s13048-024-01351-1 (PMC10837925; doi:10.1186/s13048-024-01351-1)

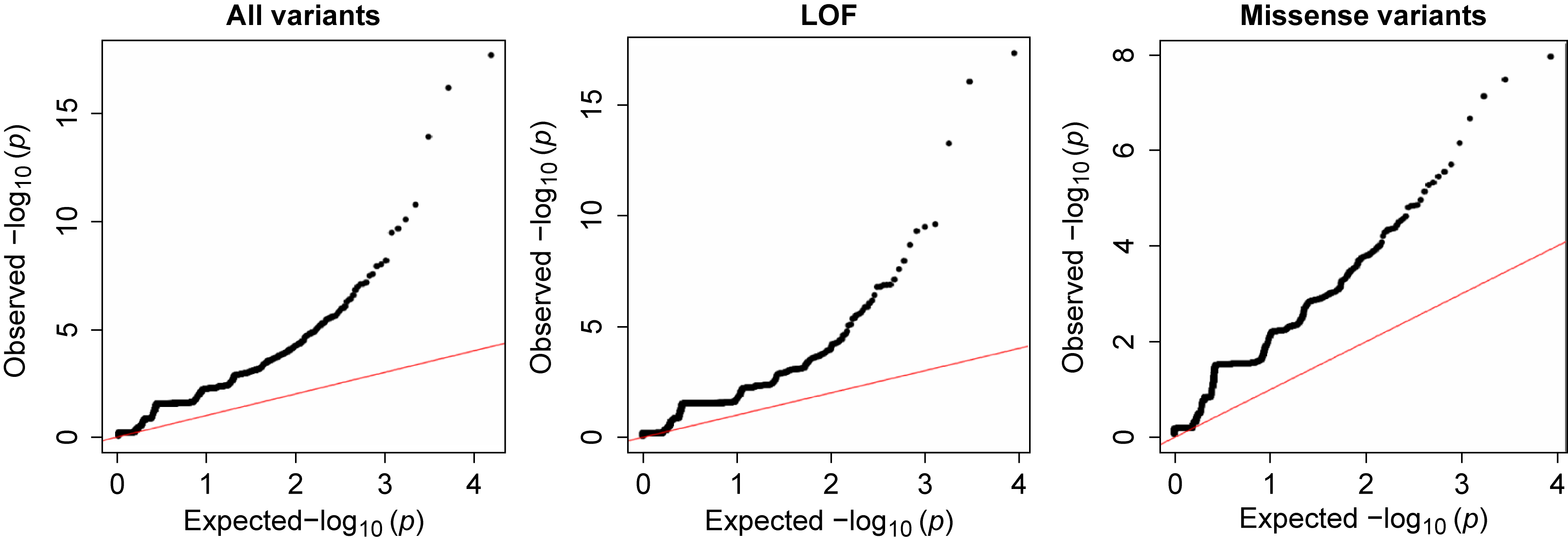

Supplement: Supplementary file 2 — Additional File 2: Figure S1. Quantile-quantile plot. LOF, loss-of-function. [file 13048_2024_1351_MOESM2_ESM.tif]

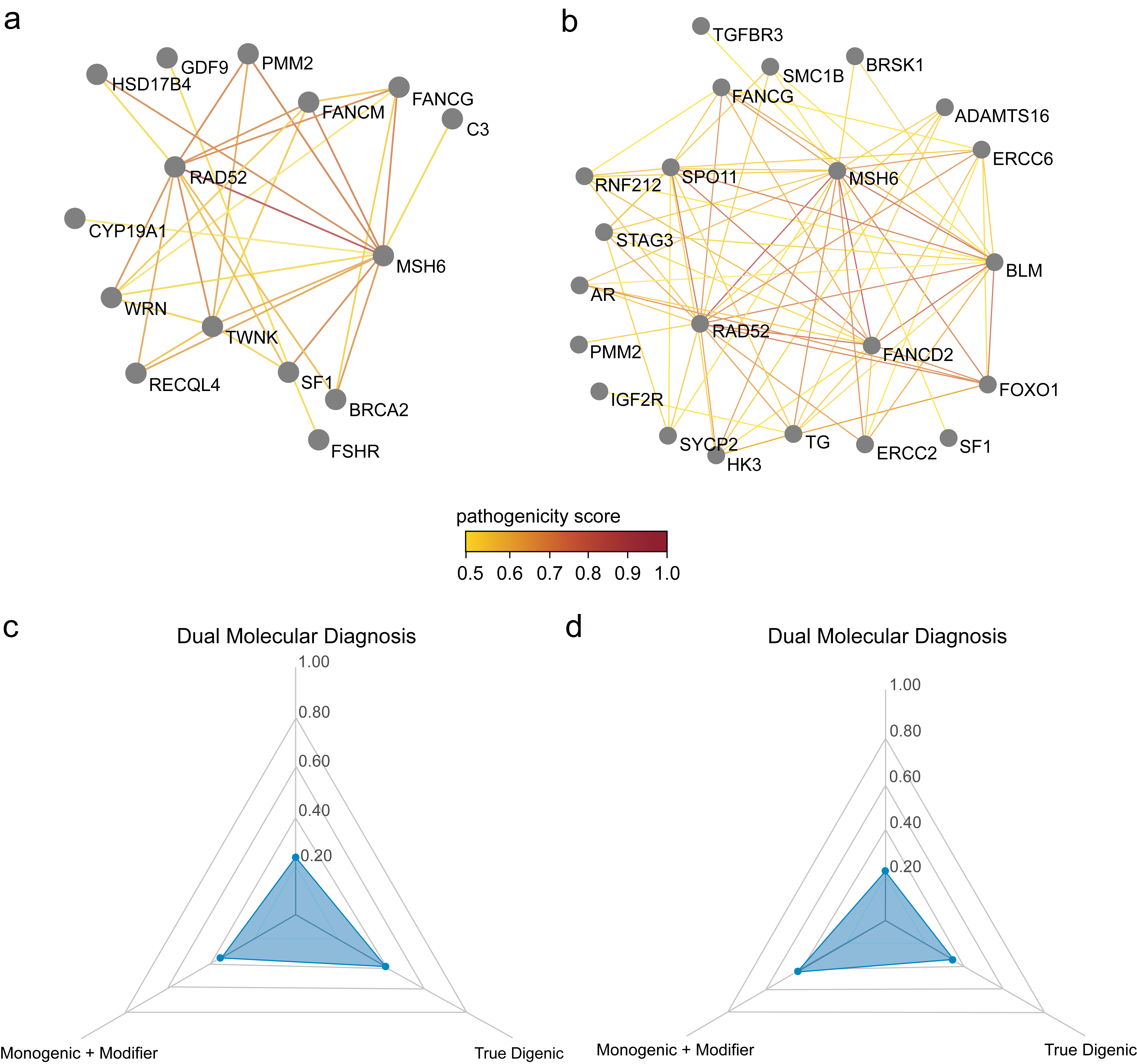

Supplement: Supplementary file 6 — Additional File 6: Figure S2. Oligogenic combination networks and radar plots. (a, b) Oligogenic combination networks for patients 64 and 66. Gene pairs are connected if they contain at least one pathogenic variant combination. Edge color: highest pathogenicity score (highest VarCoPP score) for a variant in the pair, shown from low (yellow) to high (dark red) pathogenicity scores. (c, d) Radar plots of the prediction results of the Digenic Effect for patients 64 and 66. [file 13048_2024_1351_MOESM6_ESM.tif]
