# Supplementary material for: Oligogenic basis of premature ovarian insufficiency: an observational study
Source: J Ovarian Res. 2024 Feb 3;17:32. doi: 10.1186/s13048-024-01351-1 (PMC10837925; doi:10.1186/s13048-024-01351-1)
Supplement: Supplementary file 3 — Additional File 3: TableS2. Cases involving more than one POI-related variant. [file 13048_2024_1351_MOESM3_ESM.docx]

**Additional File 3**

**Table S2**. Cases involving more than one POI-related variant.

| Sample ID | Gene list |
| --- | --- |
| 3 | *MLH1, TWNK* |
| 4 | *SOHLH1, NUP107* |
| 5 | *IGSF10, FANCM* |
| 11 | *RAD52, EIF2B2* |
| 12 | *RECQL4, GALT, FANCI, BRCA1* |
| 15 | *TP63, ERCC6, EIF2B2* |
| 16 | *RAD52, TEP1, POF1B* |
| 19 | *HFM1, TP63, AR* |
| 26 | *MSH5, MEI4, RAD52* |
| 30 | *MSH4, MSH6, IGSF10, EIF2B2* |
| 33 | *POLG2, AR* |
| 37 | *MSH6, IGSF10* |
| 39 | *HFM1, IGSF10* |
| 44 | *MLH1, RAD52, POLG* |
| 45 | *FANCD2, SPIDR, ERCC6, POF1B* |
| 47 | *HFM1, MND1, GDF9, GALT* |
| 48 | *MSH6, MLH1, AARS2* |
| 49 | *SYCP1, TP63, PMM2, MEI1, AR* |
| 54 | *MND1, AMH* |
| 58 | *MSH6, FOXL2, FANCG, BRCA1* |
| 60 | *HFM1, RAD52, NUP107* |
| 61 | *MSH4, FANCI* |
| 64 | *MSH6, TWNK, RAD52* |
| 66 | *MSH6, RNF212, RAD52* |
| 67 | *IGSF10, POF1B* |
| 73 | *MSH6, TP63, MSH5, FOXO4* |
| 78 | *IGSF10, TEP1* |
| 79 | *TG, AR, DACH2* |
| 80 | *MLH1, NUP107, BRCA1, DACH2* |
| 81 | *MND1, AARS2* |
| 84 | *POU5F1, POLG* |
| 85 | *MSH6, GDF9* |
| 89 | *MLH1, ERCC6* |

POI, premature ovarian insufficiency.
