# Supplementary material for: Oligogenic basis of premature ovarian insufficiency: an observational study
Source: J Ovarian Res. 2024 Feb 3;17:32. doi: 10.1186/s13048-024-01351-1 (PMC10837925; doi:10.1186/s13048-024-01351-1)
Supplement: Supplementary file 4 — Additional File 4: Table S3. RAD52 variants detected in patients with POI. [file 13048_2024_1351_MOESM4_ESM.docx]

**Additional File 4**

**Table S3**. *RAD52* variants detected in patients with POI

| Sample ID | Genotype | Nucleotide change | Protein change | Maximum frequency | Additional variants in POI-related genes |
| --- | --- | --- | --- | --- | --- |
| 11 | Heterozygous | NC_000012.11(NM_134424.4):c.1037C>A | p.Ser346* | 0.0168 | *EIF2B2* |
| 16 | Heterozygous | NC_000012.11(NM_134424.4):c.1037C>A | p.Ser346* | 0.0168 | *TEP1, POF1B* |
| 26 | Heterozygous | NC_000012.11(NM_134424.4):c.1037C>A | p.Ser346* | 0.0168 | *MSH5, MEI4* |
| 44 | Heterozygous | NC_000012.11(NM_134424.4):c.1037C>A | p.Ser346* | 0.0168 | *MLH1, POLG* |
| 60 | Heterozygous | NC_000012.11(NM_134424.4):c.1037C>A | p.Ser346* | 0.0168 | *HFM1, NUP107* |
| 64 | Heterozygous | NC_000012.11(NM_134424.4):c.1037C>A | p.Ser346* | 0.0168 | *MSH6, TWNK* |
| 66 | Heterozygous | NC_000012.11(NM_134424.4):c.1037C>A | p.Ser346* | 0.0168 | *MSH6, RNF212* |

POI, premature ovarian insufficiency.
