# Supplementary material for: Oligogenic basis of premature ovarian insufficiency: an observational study
Source: J Ovarian Res. 2024 Feb 3;17:32. doi: 10.1186/s13048-024-01351-1 (PMC10837925; doi:10.1186/s13048-024-01351-1)
Supplement: Supplementary file 5 — Additional File 5: Table S4. Patients heterozygous for the RAD52 and MSH6 combination. [file 13048_2024_1351_MOESM5_ESM.docx]

**Additional File 5**

**Table S4**. Patients heterozygous for the *RAD52* and *MSH6* combination

|  | Patients with the gene pair *RAD52* and *MSH6* | Odds ratio (95% confidence interval) | *P-*value |
| --- | --- | --- | --- |
| POI (*n* = 93) | 2 | 1 | / |
| Controls (*n* = 473) | 0 | / | 0.027 |

POI, premature ovarian insufficiency.
