# Supplementary material for: Oligogenic basis of premature ovarian insufficiency: an observational study
Source: J Ovarian Res. 2024 Feb 3;17:32. doi: 10.1186/s13048-024-01351-1 (PMC10837925; doi:10.1186/s13048-024-01351-1)
Supplement: Supplementary file 7 — Additional File 7: Table S5. Gene sets included in the analysis [file 13048_2024_1351_MOESM7_ESM.docx]

**Additional File 7**

**Table S5**. Gene sets included in the analysis

| 191 POI-related genes | | | | | | | |
| --- | --- | --- | --- | --- | --- | --- | --- |
| *AARS2* | *ADAMTS* | *ADAMTS16* | *ADAMTS19* | *ADAMTS9* | *AIRE* | *AMH* | *AMHR2* |
| *ANKK1* | *ANKRD31* | *AR* | *ATG7* | *ATG9A* | *ATM* | *ATPase6* | *BIRC1* |
| *BLAP75* | *BLM* | *BMP15* | *BMPR1A* | *BMPR1B* | *BMPR2* | *BNC1* | *BRCA1* |
| *BRCA2* | *BRIT1* | *BRSK1* | *C10orf2* | *C3* | *CAV1* | *CITED1* | *CITED2* |
| *CITED4* | *CLPP* | *CPEB1* | *CTIP* | *CYP17A1* | *CYP19A1* | *DACH2* | *DMC1* |
| *EIF2B2* | *EIF2B4* | *EIF2B5* | *EIF4ENIF1* | *ERCC1* | *ERCC2* | *ERCC3* | *ERCC6* |
| *ESR1* | *ESR2* | *EXO1* | *FAM175A* | *FANCA* | *FANCC* | *FANCD2* | *FANCG* |
| *FANCI* | *FANCL* | *FANCM* | *FIGLA* | *FMR1* | *FOXL2* | *FOXO1* | *FOXO1A* |
| *FOXO3* | *FOXO4* | *FSHB* | *FSHR* | *GALT* | *GDF9* | *GON1* | *GREM1* |
| *HARS2* | *HEI10* | *HELO* | *HELQ* | *HFM1* | *HK3* | *HOP2* | *HORMAD1* |
| *HSD17B4* | *HUS1* | *IGF1* | *IGF2R* | *IGSF10* | *KHDRBS1* | *LARS2* | *LHCGR* |
| *LHX8* | *MCM2* | *MCM3* | *MCM7* | *MCM8* | *MCM9* | *MEI1* | *MEI4* |
| *MEILB2* | *MLH1* | *MLH3* | *MND1* | *MRE11* | *MRPS22* | *MSH3* | *MSH4* |
| *MSH5* | *MSH6* | *NABP2* | *NANOS3* | *NBN* | *NBS1* | *NOBOX* | *NOG* |
| *NOTCH2* | *NR5A1* | *NUP107* | *Nupr1* | *OCT4* | *p63* | *PADI6* | *PCSK1* |
| *PGBD3* | *PGRMC1* | *PGRMC2* | *PMM2* | *Po1B* | *POF1B* | *POLG* | *POLG1* |
| *POLG2* | *POLR2C* | *POU5F1* | *PRDM1* | *PRIM1* | *PSMC3IP* | *RAD1* | *RAD21L* |
| *RAD50* | *RAD51* | *RAD52* | *RAD54* | *RCBTB1* | *REC114* | *REC8* | *RECQL2* |
| *RECQL3* | *RECQL4* | *RNF212* | *SALL4* | *SF1* | *Sgo1* | *SGO2* | *SMC1B* |
| *SMC3* | *SOHLH1* | *SOHLH2* | *SOX3* | *SPATA2* | *SPIDR* | *SPO11* | *SRD5A1* |
| *STAG3* | *STAR* | *SYCE1* | *SYCE2* | *SYCE3* | *SYCP1* | *SYCP2* | *SYCP2L* |
| *SYCP3* | *TEP1* | *TERC* | *TEX11* | *TG* | *TGFBR1* | *TGFBR3* | *TGIF2LX* |
| *TLK1* | *TNF* | *TopoⅢα* | *TP1* | *TP63* | *TRC3* | *TRIM37* | *TWNK* |
| *UIMC1* | *UMC1* | *WRN* | *WT1* | *XRCC2* | *XRCC4* | *ZNF830* |  |

POI, premature ovarian insufficiency.
