# Supplementary material for: Oligogenic basis of premature ovarian insufficiency: an observational study
Source: J Ovarian Res. 2024 Feb 3;17:32. doi: 10.1186/s13048-024-01351-1 (PMC10837925; doi:10.1186/s13048-024-01351-1)
Supplement: Supplementary file 8 — Additional File 8: Table S6 Four gene sets with biological functions related to POI. [file 13048_2024_1351_MOESM8_ESM.docx]

**Additional File 8**

**Table S6**. Four gene sets with biological functions related to POI.

| **Meiosis and DNA damage repair** | | | | | | | |
| --- | --- | --- | --- | --- | --- | --- | --- |
| *ATM* | *BRWD1* | *CDC25B* | *CDK2* | *CKS2* | *CPEB1* | *CYP26B1* | *DMC1* |
| *ERCC1* | *ERCC2* | *CBSPGBD3* | *FANCA* | *FANCC* | *FANCG* | *FANCL* | *GJA4* |
| *GPR3* | *HFM1* | *HSF2* | *MCM8* | *MCM9* | *MEI1* | *MLH1* | *MLH3* |
| *MOS* | *MSH4* | *MSH5* | *NOS3* | *NUP107* | *PMS2* | *PSMC3IP* | *RAD51B* |
| *REC8* | *SGOL2* | *SMC1B* | *SPO11* | *STAG3* | *SYCE1* | *SYCP1* | *SYCP2* |
| *SYCP3* | *TOP3B* | *TRIP13* | *UBB* | *UBR2* | *SYCE2* | *SYCE3* | *TEX11* |
| *SMC1B* | *RAD21L* | *SMC3* | *Sgo1* | *Sgo2* | *FANCM* | *RECQL2* | *RECQL3* |
| *RECQL4* | *RAD1* | *BLM* | *TopoⅢα* | *BLAP75* | *MRE11* | *RAD50* | *NBS1* |
| *REC114* | *ANKRD31* | *EXO1* | *HELO* | *ZNF830* | *FANCD2* | *FANCI* | *FAM175A* |
| *UMC1* | *TLK1* | *HORMAD1* | *MEILB2* | *MEI4* | *CTIP* | *PoIB* | *HOP2* |
| *MND1* | *BRCA2* | *PRIM1* | *SPIDR* | *RAD51* | *RAD52* | *RAD54* | *BRIT1* |
| *HUS1* | *SPATA2* | *NABP2* | *MSH3* | *MSH6* | *MCM2* | *MCM3* | *MCM7* |
| *ERCC6* | *ERCC3* | *PGBD3* | *MLH3* | *XRCC4* | *RNF212* | *HEI10* | *SGO2* |
| *SGO1* | *SMC1B* | *REC8* | *STAG3* | *MRE11* | *RAD50* | *NBS1* |  |
| **Gonadal formation** | | | | | | | |
| *CBX2* | *CTNNB1* | *DHH* | *EMX2* | *FGF9* | *FST* | *GATA4* | *LHX9* |
| *NR0B1* | *NR5A1* | *RSPO1* | *SOX9* | *SOX8* | *SRY* | *WNT4* | *WT1* |
| *ZFPM2* |  |  |  |  |  |  |  |
| **Ovarian development** | | | | | | | |
| *AMH* | *AMHR2* | *BCL2* | *BCL2L2* | *BMP15* | *BMP4* | *BMP8B* | *BMPR1B* |
| *BMPR2* | *CDKN1B* | *CYP11A1* | *CYP17A1* | *CYP19A1* | *DAZL* | *DIAPH2* | *DND1* |
| *EIF2B2* | *EIF2B5* | *eIF4ENIF1* | *FIGLA* | *FMN2* | *FOXL2* | *FOXO1* | *FOXO3* |
| *FOXO4* | *FSHR* | *GDF9* | *GJA4* | *INHA* | *INHBA* | *INHBB* | *INSL3* |
| *KIT* | *KITLG* | *LHCGR* | *LHX8* | *NANOS1* | *NANOS2* | *NANOS3* | *NBN* |
| *NOBOX* | *PGRMC1* | *POF1B* | *POLR3H* | *POR* | *POU5F1* | *PDGFRA* | *PRDM1* |
| *PTEN* | *SMAD1* | *SMAD4* | *SMAD5* | *SOHLH1* | *SOHLH2* | *SOX3* | *STAR* |
| *STRA8* | *TCF21* | *TGFBR3* | *TIAL1* | *UBE3A* | *ZFX* |  |  |
| **Signaling molecules and transcription factors** | | | | | | | |
| *ADAMTS* | *ADAMTS16* | *ADAMTS19* | *ADAMTS9* | *AR* | *ATPase6* | *BIRC1* | *BMP15* |
| *CITED1* | *CITED2* | *CITED4* | *CLPP* | *CPEB1* | *DIAPH2* | *ESR1* | *FIGLA* |
| *FMR1* | *FOXL2* | *FOXO1* | *FOXO1A* | *FOXO3* | *FOXO4* | *FSHR* | *GDF9* |
| *GON1* | *GREM1* | *HAX1* | *KHDRBS1* | *LHX8* | *NANOS3* | *NOBOX* | *Nupr1* |
| *OCT4* | *p63* | *PGRMC1* | *PGRMC2* | *POF1B* | *POLG1* | *POLG2* | *POLR2C* |
| *PRDM1* | *R-spondin2* | *SALL4* | *SF1* | *SOHLH1* | *SOHLH2* | *SOX3* | *TEP1* |
| *TERC* | *TGIF2LX* | *TP1* | *TP63* | *TRC3* | *TWNK* | *Wnt3* | *Wnt4a* |
| *WT1* |  |  |  |  |  |  |  |

POI, premature ovarian insufficiency.
